# Ischaemic Preconditioning Protects Cardiomyocytes from Anthracycline-Induced Toxicity via the PI3K Pathway

**DOI:** 10.1007/s10557-018-6793-y

**Published:** 2018-05-15

**Authors:** Angshuman Maulik, Sean M. Davidson, Izabela Piotrowska, Malcolm Walker, Derek M. Yellon

**Affiliations:** The Hatter Cardiovascular Institute, 67 Chenies Mews, London, WC1E 6HX UK

**Keywords:** Anthracyclines, Preconditioning, Cardiotoxicity, PI-3kinase, Cardioprotection

## Abstract

**Purpose:**

Anthracyclines cause chronic irreversible cardiac failure, but the mechanism remains poorly understood. Emerging data indicate that cardiac damage begins early, suggesting protective modalities delivered in the acute stage may confer prolonged benefit. Ischaemic preconditioning (IPC) activates the pro-survival reperfusion injury salvage kinase (RISK) pathway which involves PI3-kinase and MAPK/ERK1/2.

**Methods:**

We investigated whether simulated IPC (sIPC), in the form of a sublethal exposure to a hypoxic buffer simulating ischaemic conditions followed by reoxygenation, protects primary adult rat cardiomyocytes against anthracycline-induced injury. PI3-kinase and MAPK/ERK1/2 were inhibited using LY294002, and PD98059. The role of reactive oxygen species (ROS), mitochondrial membrane potential (Δψ_m_) and mitochondrial permeability transition pore (mPTP) were also investigated in doxorubicin-treated cells. We further examined whether sIPC protected HeLa cancer cells from doxorubicin-induced death.

**Results:**

sIPC protected cardiomyocytes against doxorubicin-induced death (35.4 ± 1.7% doxorubicin vs 14.7 ± 1.5% doxorubicin + sIPC; *p* < 0.01). This protection was abrogated by the PI3-kinase inhibitor, LY294002, but not the MAPK/ERK1/2 inhibitor, PD98059. A ROS scavenger failed to rescue cardiomyocytes from doxorubicin toxicity, and no significant influence on Δψ_m_ or mPTP opening was identified after subjecting cells to a doxorubicin insult. Importantly, sIPC did not protect HeLa cancer cells from doxorubicin-induced death.

**Conclusion:**

sIPC is able to protect cardiomyocytes against anthracycline injury via a pathway involving PI3-kinase. This mechanism appears to be independent of ROS, changes to Δψ_m,_ and mPTP. Further investigation of the mechanism of sIPC-induced protection against anthracycline-injury is warranted.

## Introduction

Anthracyclines, such as doxorubicin, are a group of anthracenedione antibiotics with cytotoxic potential. They form an integral part of chemotherapy against a multitude of cancers, including solid organ tumours, childhood cancers, as well as haematological malignancies [1]. Unfortunately, the scope of anthracycline-therapy is significantly limited by cardiotoxicity. Acutely, this may manifest as transient contractile dysfunction, inflammatory heart diseases (such as myocarditis or pericarditis), conduction abnormalities or altered myocardial repolarisation. Chronically, anthracycline-based chemotherapy can result in irreversible heart failure manifesting as dilated cardiomyopathy (DCM) and refractory congestive cardiac failure (CCF). However, emerging data suggest that cardiac damage begins early and may in fact be a continuum, implying that the arbitrary division into acute and chronic damage may be artificial [2]. Cardioprotective modalities effective against acute injury may therefore provide prolonged benefit.

The mechanisms of anthracycline-mediated cardiac injury remain incompletely understood. Reactive oxygen species (ROS)-mediated oxidative stress, iron-overload mediated toxicity, alterations of mitochondrial transmembrane potential (ΔΨ_m_) and induction of the mitochondrial permeability transition pore (mPTP) opening have all been postulated to be involved in the pathological mechanism [3]. However, multiple interventions including ROS-scavenging with N-acetyl cysteine (NAC) or iron-chelation with dexrazoxane have failed to translate clinically to providing meaningful protection against cardiotoxicity caused by doxorubicin [4]. Of note however, doxorubicin has been shown to inhibit a number of pro-survival signalling cascades. For example, dephosphorylation of PI3-kinase (PI3K)/Akt after doxorubicin exposure may lead to activation of apoptotic pathways [5]. Conversely, activation of PI3K signalling has been demonstrated to ameliorate doxorubicin-induced cardiomyopathy in vivo [6]. Other experiments have suggested that the MAPK/ERK1/2 pathway can protect against anthracycline-induced cardiotoxicity [7–9].

It has been known for many years,that brief periods of ischemia followed by reperfusion can protect the heart against a subsequent prolonged bout of ischemia. This is known as ischemic preconditioning (IPC) [10]. A similar phenomenon can be observed in vitro, by simulating IPC using brief exposure to a hypoxic buffer simulating ischaemic conditions, followed by reoxygenation. This form of IPC protects cardiomyocytes against subsequent hypoxic cell death. Evidence to date implicates a complex kinase signalling cascade in the mechanism of IPC, including activation of PI3-kinase (PI3K), protein kinase C (PKC) and MAP kinase pathways, which are collectively termed the “reperfusion injury salvage kinase” (or RISK) pathway [11]. These signalling pathways converge on mitochondria to protect the cardiomyocytes by inhibiting opening of the MPTP [10]. Crucially, PI3K inhibition has been shown to abrogate IPC-mediated protection [12–15].

It has been shown using an isolated, perfused rat heart model that IPC can prevent some of the loss of function that occurs when an anthracycline (2 mg/ml epirubicin in this case) is added to the perfusate for 20 min [16]. However, whether IPC can directly protect cardiomyocytes against anthracycline toxicity has not been examined. Given the similar pathways of cardiac injury induced by doxorubicin and ischemia-reperfusion injury, we investigated the hypothesis that simulated IPC (sIPC) can protect cardiomyocytes against anthracycline-toxicity in an in vitro model using primary adult rat cardiomyocytes. It was also important to establish whether such a cardioprotective modality would protect a cancer cell line from doxorubicin-induced cell death, for which we used HeLa cells, a cervical cancer cell line. For these studies, we chose to use an acute, in vitro model of 18 h doxorubicin exposure, since doxorubicin toxicity has been shown to occur early and to be cumulative [17, 18].

## Materials and Methods

### Animals

Male adult Sprague-Dawley rats (150–450 g weight) were obtained from UCL biological services unit. All animals were housed in a temperature-, humidity- and light cycle-controlled environment. All animal experiments were carried out in accordance with the UK Home Office Guide on the Operation of Animal (Scientific Procedures) Act of 1986.

### Isolation of Ventricular Cardiomyocytes

Ventricular cardiomyocytes were isolated from adult male Sprague-Dawley rats as described previously [19] but with omission of heparin whilst administering terminal anaesthesia with pentobarbital sodium (55 mg/kg) injected i.p. After isolation, cells were cultured in M199 (invitrogen) supplemented with creatine (5 mM), carnitine (2 mM) and taurine (5 mM) and streptomycin/penicillin. Cell isolations yielding > 20% death after 18 h under basal conditions were not used.

### Doxorubicin Treatment and Simulated Ischaemic Preconditioning

Cells were preconditioned by exposing them to hypoxia for 30 min in a buffer simulating tissue ischaemia, followed by 10 min reoxygenation in a buffer simulating reperfusion, prior to exposure to doxorubicin. Hypoxia was induced in an airtight hypoxic chamber using a buffer containing 127.8 mM NaCl, 14.8 mM KCl, 1.2 mM KH_2_PO_4_, 1.2 mM MgSO_4_, 2.2 mM NaHCO_3_, 1.0 mM CaCl_2_ and 10 mM sodium-lactate. The buffer was gassed with 95% N_2_/5% CO_2_ immediately before use and the pH was adjusted to 6.4 at 37 °C. Reoxygenation was carried out in a normoxic buffer containing 10 mM glucose, 118 mM NaCl, 1.2 mM KH_2_PO_4_, 1.2 mM MgSO_4,_ 22 mM NaHCO_3_ and 1.0 mM CaCl_2,_ gassed with carbogen and the pH adjusted at 37 °C to pH 7.4 before use [20]. Preconditioned cardiomyocytes were incubated with the indicated concentrations of doxorubicin for 18 h. Control (non-preconditioned) cardiomyocytes were incubated in normoxic buffer for 40 min before replacing the buffer with M199 containing doxorubicin. HeLa cells were treated similarly but using a concentration of 2 μM doxorubicin since they are innately more sensitive to doxorubicin.

To investigate the components of the RISK pathway, cardiomyocytes were subjected to sIPC and doxorubicin in the presence of 10 μM LY294002 (Sigma Aldrich) to inhibit PI3K/Akt pathway, or 30 μM PD98059 (Sigma Aldrich) to inhibit MAPK/ERK1/2, respectively.

### Western Blot Analyses

Cells were lysed by boiling in SDS-PAGE sample loading buffer (1% SDS, 10% glycerol, 10 mM Tris-Cl, pH 6.8, 1 mM EDTA). As positive control for Akt phosphorylation, cardiomyocytes were treated with 100 nM insulin. The samples were resolved by SDS-PAGE in 10% acrylamide gel. Phosphorylated and non-phosphorylated Akt was compared to the content of the housekeeping protein glyceraldehyde 3-phosphate dehydrogenase (GAPDH). To evaluate phosphorylation status of MAPK/ ERK1/2, phosphorylated and non-phosphorylated ERK1/2 was compared to the content of the housekeeping protein alpha-tubulin.

Primary antibodies used were anti-tAkt (Cell Signaling Technology, Inc. Cat. no. 2920) anti-pAkt (Phospho-Serine 473, Cell Signalling Technology, Inc. Cat. no. 4060), anti-tERK1/2 (Cell Signalling Technology, Inc. Cat. no. 9107), anti- pERK1/2 (Phospho-Threonine 202/Phospho-Tyrosine 204, Cell Signalling Technology, Inc. Cat. no. 4370), anti-GAPDH (abcam, Cat no. ab9485) and anti-alpha tubulin (abcam, Cat no. ab4074) All primary antibodies were used at a dilution of 1:1000. Following overnight incubation, the membrane was washed and probed with secondary antibodies using standard Western blot protocol.

### Investigation of Reactive Oxygen Species

Cardiomyocytes were incubated with 7.5 μM doxorubicin, in the presence or absence of N-acetyl cysteine (250 μM NAC, Thermo Fisher Scientific). As positive control, cardiomyocytes were incubated with 1 μM H_2_O_2_ (Sigma-Aldrich). Cell-death was evaluated at the end of experiment.

### Measurement of Mitochondrial Transmembrane Potential

Cardiomyocytes were cultured in M199 and 7.5 μM doxorubicin added as indicated. Thirty nanomolars tetramethyl rhodamine methyl ester (TMRM, Thermo Fisher) was added for 30 min in Tyrode’s solution (137 mM NaCl, 2.7 mM KCl, 1 mM MgCl2, 1.8 mM CaCl2, 0.2 mM Na_2_HPO_4_, 12 mM NaHCO_3_, 5.5 mM D-glucose, pH 7.4), before transferring to dye-free Tyrode’s solution and analysed by confocal microscopy. Transmembrane potential was measured in a Leica SP5 confocal microscope using 10% He-Ne Laser at 543 nm excitation wavelength, and intensity of the emission at 581–596 nm was recorded. Images were analysed using ImageJ software.

### Measurement of Mitochondrial Permeability Pore Induction

Cardiomyocytes were incubated with 7.5 μM doxorubicin dissolved in M199 for 18 h, before loading the cells with 3 μM TMRM in solution in Tyrode’s buffer. The medium was replaced with dye-free Tyrode’s buffer, and time taken to mPTP opening determined as described previously [19]. One micromolar cyclosporine A (CsA, Merck Millipore) an agent known to inhibit pore opening was used as a positive control.

### Assessment of Cell Death

At the end of cell-survival experiments, 5 μg/ml propidium iodide (PI, Thermo Fisher) was added prior to microscopic analysis. Representative images were recorded using a Nikon Eclipse TE200 microscope and analysed using ImageJ. Cardiomyocyte-death was assessed on the basis of displaying PI-positive nuclear staining or a terminally damaged morphology [21]. HeLa cell death was based purely on PI-positive staining.

### Statistical Analysis

All data were analysed using Graphpad prism (version 5.0). Values are expressed as mean ± SEM. Analysis was carried out using two-way ANOVA, followed by post hoc comparison test when significant, using Bonferoni correction for multiple comparisons. Differences were considered significant if *p* values were determined to be *p* < 0.05. * represents *p* < 0.05; ** *p* < 0.01; *** *p* < 0.001.

## Results

### Hypoxic Preconditioning Protects Cardiomyocytes Against Doxorubicin-Induced Injury

**Fig. 1:**
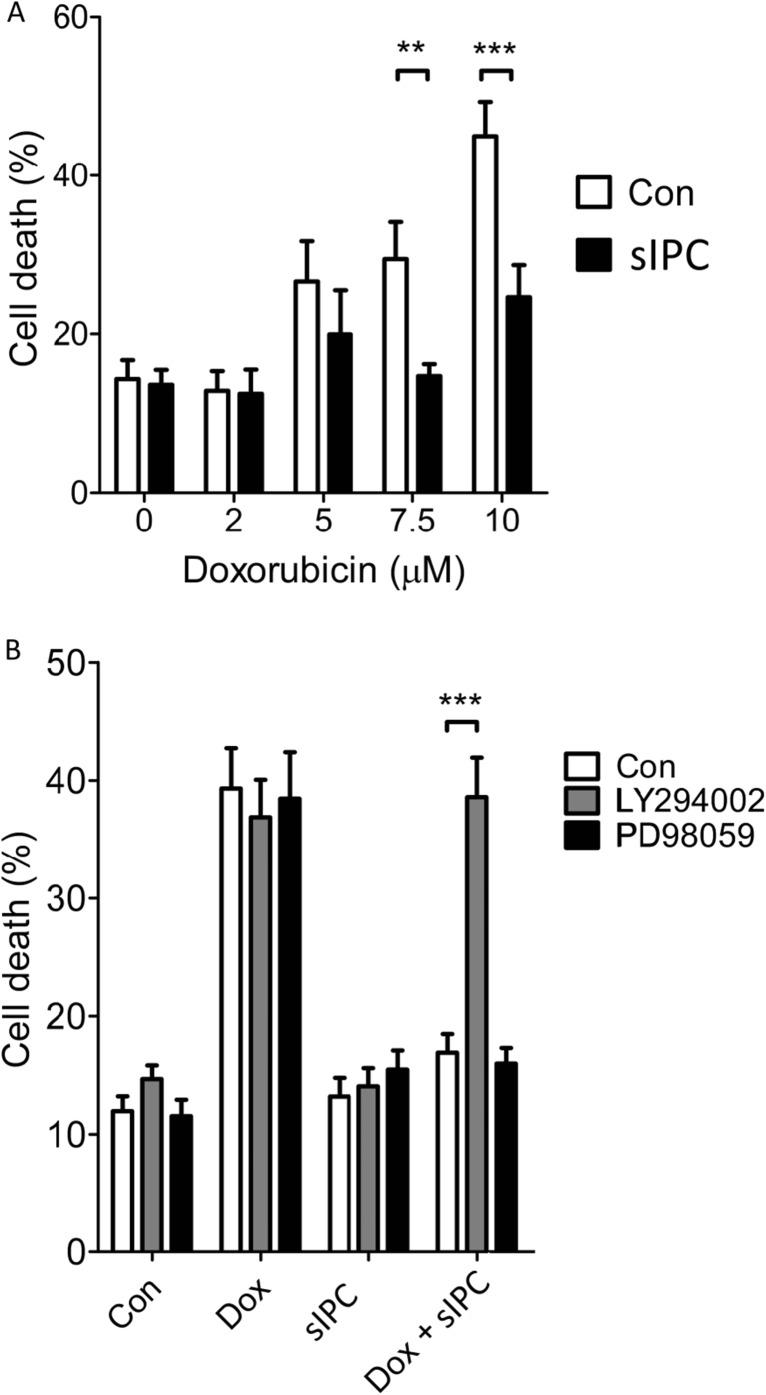
**a** The protective effect of simulated ischaemic preconditioning (sIPC) against a range of doxorubicin (Dox) concentrations. Primary adult rat cardiomyocytes were subject to 18 h Dox treatment at the indicated concentrations, with or without preceding simulated ischaemic preconditioning (sIPC). sIPC prevented the increase in cell death from 7.5 or 10 μM Dox (** *p* < 0.01, *** *p* < 0.001 *n* = 5). **b** Inhibiting PI3K/Akt but not MAPK/ERK1/2 eliminated sIPC-mediated protection against cell death caused by 18 h exposure to 7.5 μM doxorubicin. Cells were treated with LY294002 to inhibit PI3K/Akt or PD98059 to inhibit MAPK/ERK1/2, throughout exposure to sIPC and Dox as in panel **a**. (*** *p* < 0.001 *n* = 5)

We first performed a dose-response experiment to determine the lethal concentration of 18 h doxorubicin exposure on primary adult rat cardiomyocytes. After doxorubicin treatment, cell-death was significantly higher in cells exposed to 7.5 and 10 μM doxorubicin (Fig. 1a). Next, we simulated IPC (sIPC) in vitro by subjecting the cells to hypoxia and reoxygenation and determined whether this protected the cells from death. sIPC prevented doxorubicin from significantly increasing cell death at both 7.5 and 10 μM doxorubicin (Con 15.6 ± 1.1% vs 7.5 μM Dox 37 ± 4.9% *p* < 0.01; Con vs 10 μM Dox 43.1 ± 4.6%, *p* < 0.001, *n* = 5) (Fig. 1a). Based on these results, subsequent experiments were carried out using 7.5 μM doxorubicin over a treatment period of 18 h.

We next investigated the role of the RISK pathway in sIPC-mediated protection against doxorubicin-toxicity. On their own, LY294002 or PD98059 (antagonists of PI3-kinase and ERK1/2 respectively) did not affect the levels of cell death in control cells or those treated with doxorubicin (Fig. 1b). sIPC-mediated protection against doxorubicin was unaffected by the presence of the ERK inhibitor, PD98059. However, the PI3-kinase inhibitor, LY294002, abolished protection by sIPC (Dox sIPC 16.9 ± 1.5%; Dox sIPC+PD 15.9 ± 1.3%; Dox sIPC+LY 38.5 ± 3.3%; *p* < 0.05, *n* = 5) (Fig. 1b).

**Fig. 2:**
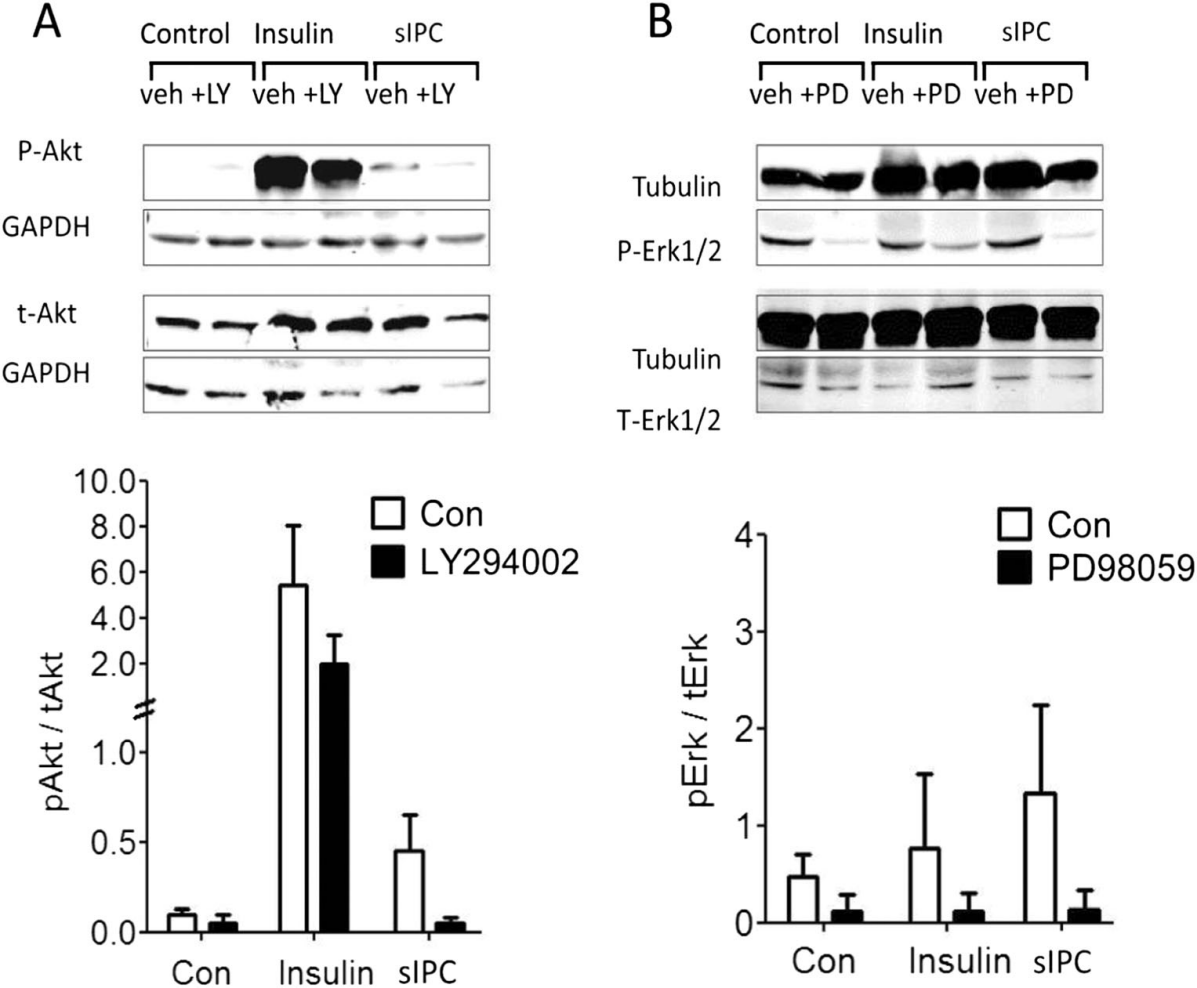
**a** Western blot analysis demonstrated that sIPC activated PI3K/Akt in cardiomyocytes, and that LY294002 (LY) inhibited this activation. Insulin, used as a positive control, strongly activated PI3K/Akt, and this was decreased by LY294002 treatment (*n* = 3). **b** Western blot analyses for MAPK/ERK1/2 similarly revealed increased phosphorylation in response to sIPC and to insulin respectively, which was abrogated by treatment with PD98059 (*n* = 3)

Western blot analysis was used to confirm the activation of the RISK pathway by sIPC in this experimental model. This confirmed that Akt phosphorylation at Ser 473 was increased after sIPC and that phosphorylation was inhibited by LY294002. Insulin, which strongly activates PI3K kinase activity, was used as a positive control for Akt phosphorylation. Insulin treatment strongly increased Akt phosphorylation (Fig. 2a). Similarly, phosphorylation of ERK1/2 at Thr 202/Tyr 204 was observed after treatment with cardiomyocytes with sIPC (Fig. 2b). Phosphorylation of ERK1/2 was inhibited in the presence of PD98059 (Fig. 2b).

### Doxorubicin-Induced Cardiomyocyte Death Is Independent of ROS-Production

**Fig. 3:**
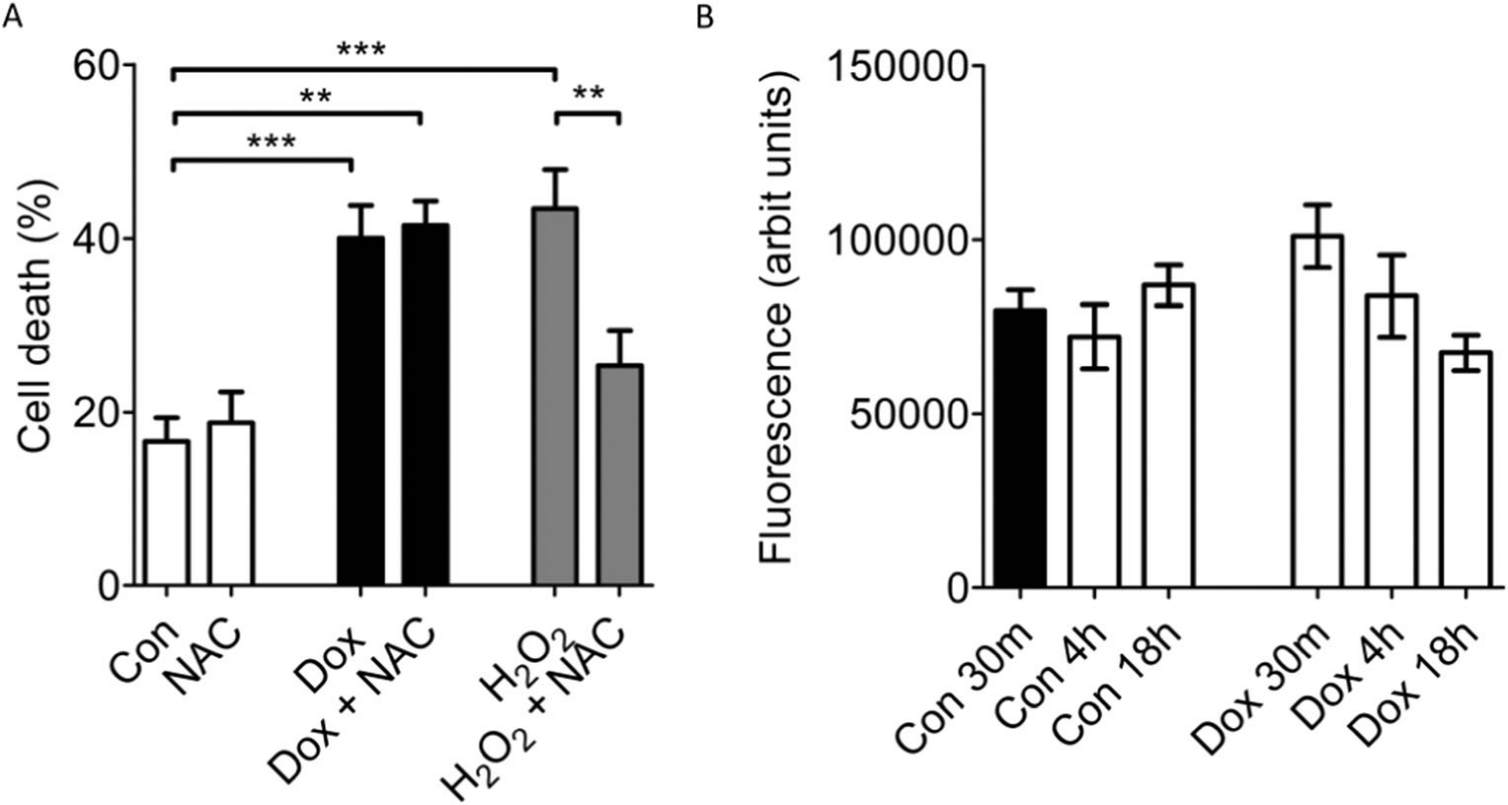
**a** A ROS scavenger (NAC) did not affect Dox-induced cardiomyocyte-death. Cardiomyocytes were exposed to 18 h 7.5 μM Dox or 1 μM H_2_O_2_ in the presence or absence of 7.5 μM NAC, prior to analysis of cell death. NAC rescued cardiomyocytes from H_2_O_2_ toxicity, but had no effect on Dox-induced death (*n* = 3, ** *p* < 0.01, *** *p* < 0.001)

Part of the mechanism by which IPC is known to protect cardiomyocytes from ischemia and reperfusion injury is by limiting ROS production and preventing mPTP opening [19, 22]. We therefore sought to understand the role for ROS and mitochondria in doxorubicin-induced death. We first examined whether a ROS scavenger was cardioprotective in our model of doxorubicin-induced toxicity. Although NAC was able to protect cardiomyocytes against 1 μM H_2_O_2_ (H_2_O_2_ 43.4 ± 4.5% vs H_2_O_2_+NAC 25.3 ± 4%; *p* < 0.01, *n* = 3) (Fig. 3a), it had no effect on doxorubicin-induced cell death (Dox 40 ± 3.7%; vs Dox NAC 41.4 ± 2.8%; *n* = 3) (Fig. 3a). NAC alone did not affect cell-death (Con 16.6 ± 2.7% vs Con NAC, 18.7 ± 3.7%) (Fig. 3a).

### Doxorubicin-Induced Toxicity Is Independent of ΔΨ_m_ and the Induction of the mPTP

**Fig. 4:**
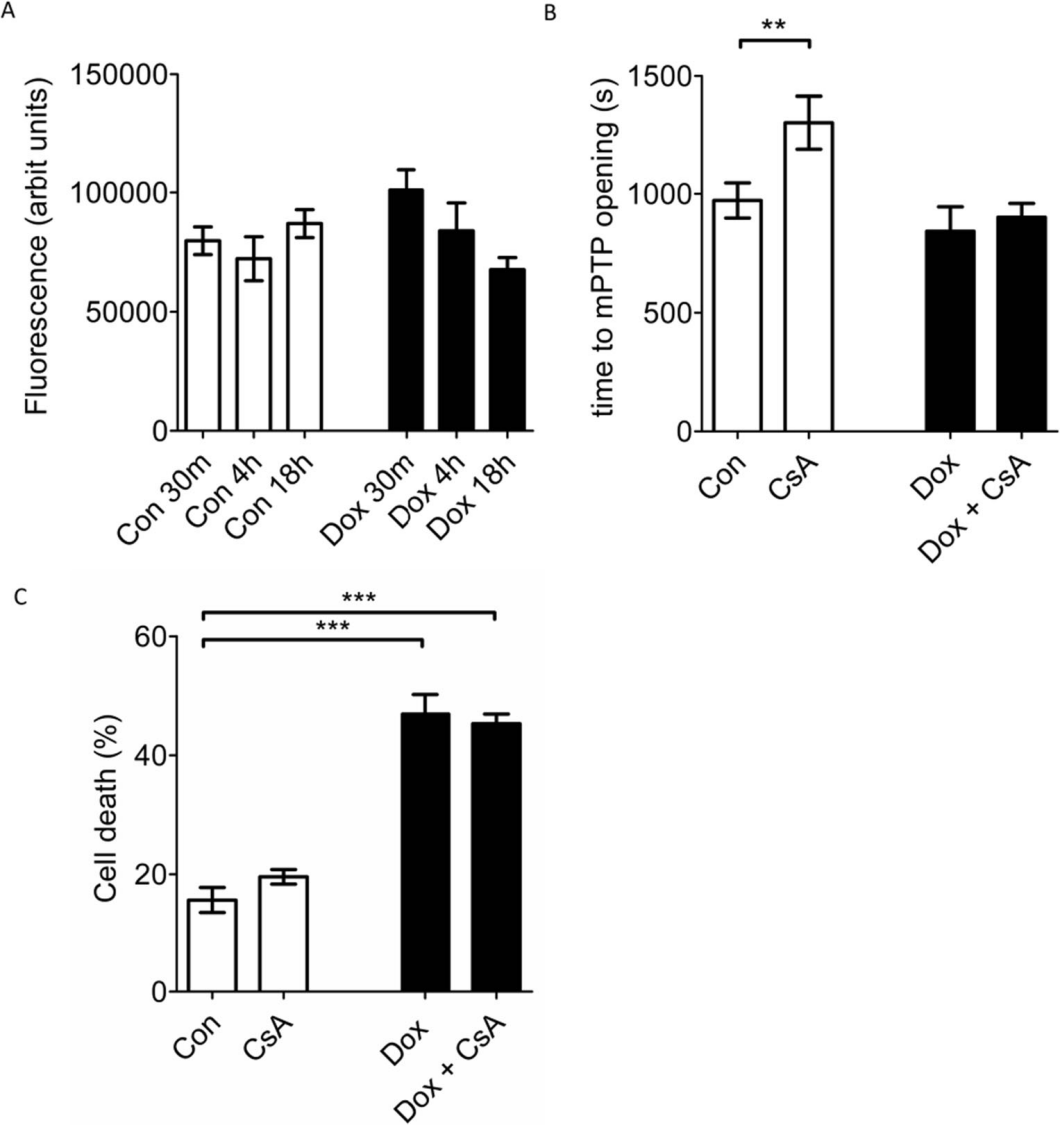
**a** Exposure to doxorubicin did not alter the sensitivity of the mPTP to oxidative stress. Cardiomyocytes were exposed to 1 μM CsA or 7.5 μM Dox for 18 h, before loading with a photo-sensitising concentration of 3 μM TMRM and subjecting cells to laser-induced oxidative stress. The time to mPTP opening was measured and was significantly increased by CsA in control cells (CsA) but not in those cells that had been exposed to Doxorubin (Dox + CsA) (*p* < 0.05, *n* = 7). **b** 1 μM CsA did not protect cardiomyocytes against exposure to 18 h 7.5 μM Dox. Cell death was measured at the end of 18 h with vehicle (Con) or Dox (*p* < 0.05, *n* = 5)

We next investigated whether mPTP opening is involved in doxorubicin-induced cardiomyocyte death in our model. We used a previously validated, laser-induced toxicity model in cardiomyocytes, which is based on the photo-oxidation of the dye TMRM in the mitochondria [23]. Before using this model, we confirmed that the mitochondrial transmembrane potential (ΔΨ_m_) (and hence mitochondrial TMRM accumulation) was unchanged in Dox-treated cells (data not shown). We then investigated whether doxorubicin treatment increases the sensitivity of the mPTP to opening. After 18 h doxorubicin treatment, no significant differences were seen in mPTP sensitivity, as measured by the time taken until mPTP opening occurred (Dox 842 ± 103 s, vs control 972 ± 74 s, *p* = 0.4, *n* = 7) (Fig. 4a). In a control group, 1 μM CsA (used as a positive control) protected against mPTP induction in untreated cardiomyocytes (CsA 1304 ± 111 s, vs Con 901 ± 58 s) (*p* < 0.01, *n* = 7), although it did not protect in cardiomyocytes subjected to doxorubicin (Fig. 4a).

In order to confirm that prevention of mPTP opening was not protective against doxorubicin-induced cell death, isolated cardiomyocytes were subjected for 18 h to 7.5 μM doxorubicin alone or in the presence of 1 μM CsA before evaluating cell death. CsA failed to protect cardiomyocytes against doxorubicin-induced cell-death (Dox, 46.9 ± 3.3% vs Dox CsA 45.3 ± 1.6%, *p* = 0.9, *n* = 5) (Fig. 4b).

### Preconditioning Does Not Protect a Cancer Cell Line from Doxorubicin

**Fig. 5:**
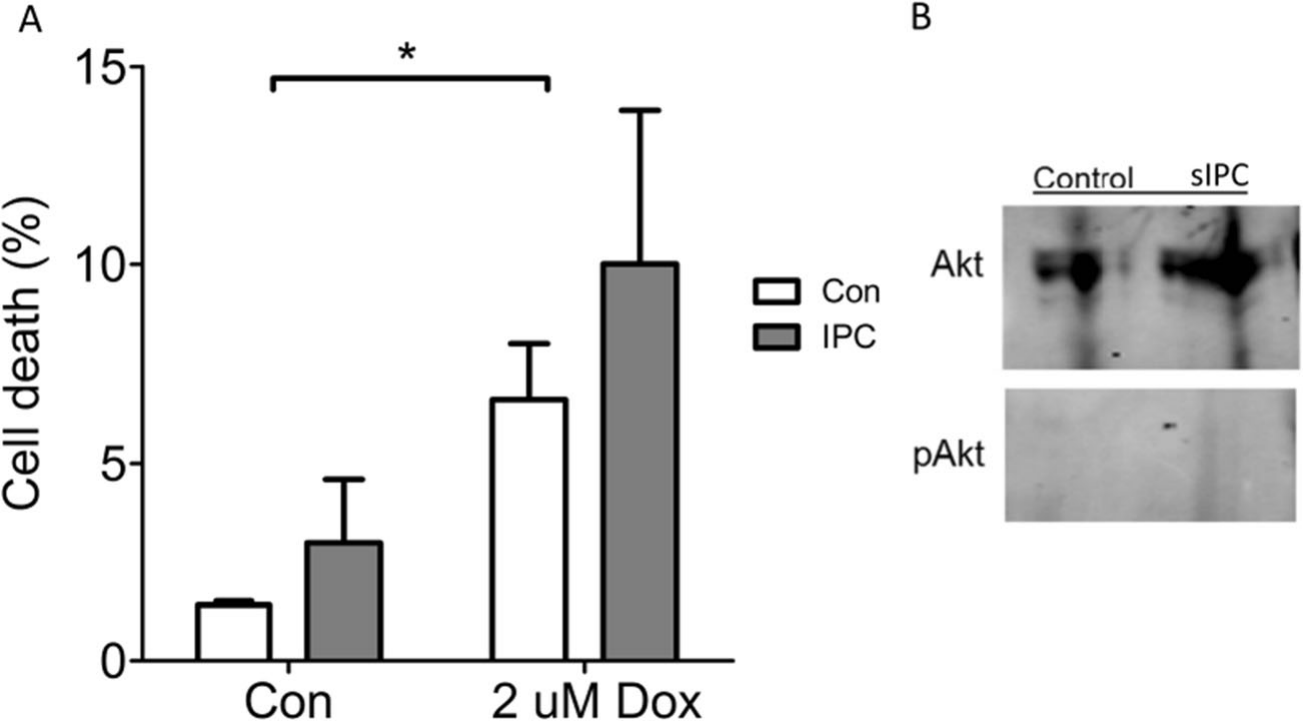
**a** 2 μM doxorubicin (Dox) increased cell death in HeLa cancer cells significantly, but preconditioning did not protect them (*n* = 5). **b** Akt was not phosphorylated in response to sIPC in HeLa cells

In order to determine whether preconditioning could protect a cancer cell line against doxorubicin, we used HeLa cervical cancer cells, which are sensitive to 2 μM doxorubicin. This dose of doxorubicin caused an increase in cell death from 1.6 ± 0.3% to 6.7 ± 1.7% after 18 h (Fig. 5a). sIPC did not protect HeLa cells against this injury but paradoxically increased cell death to 3.2 ± 2.6% in control and 10.4 ± 3.8% in those treated with doxorubicin (*n* = 5) (Fig. 5a). Furthermore, sIPC did not increase phosphorylation of Akt in HeLa cells (Fig. 5b).

## Discussion

In this study, we show for the first time that the phenomenon of ischaemic preconditioning directly protects isolated cardiomyocytes against doxorubicin-induced cell death. This protection appears to be dependent on PI3K/Akt, as confirmed by first demonstrating that sIPC increases Akt phosphorylation in cells, and secondly that both Akt phosphorylation and protection against Dox are abrogated by the presence of the PI3K inhibitor LY294002. Although sIPC increased MAPK/ERK1/2 phosphorylation, and this was abrogated by PD98059, this inhibitor did not affect sIPC protection against Dox.

Despite previous studies implicating ROS and mitochondrial damage in doxorubicin cardiotoxicity, these mechanisms did not appear to be involved in cardiotoxicity in our experimental model, leading us to conclude sIPC-mediated protection from doxorubicin-cardiotoxicity, we observed in our study, is independent of these pathways. Importantly, sIPC did not protect HeLa cancer cells from doxorubicin-induced death.

Doxorubicin has previously been shown to be toxic to cardiomyocytes in a number of different models including neonatal rat ventricular myocytes, immortalised cardiomyoblasts and cardiomyocyte-derived cell-lines [24, 25]. Treatment protocols and experimental design differ among the described models, and doses ranged from those within the physiological steady-state concentrations (in the range of 0.5–2 μM), to supraphysiological concentrations up to 27 μM [26]. For comparison, the peak plasma concentration is typically in the range of 5 μM in humans [1]. In our study, we used primary adult rat ventricular myocytes to investigate doxorubicin-induced injury. Since these are non-dividing terminally differentiated cells, the model more accurately replicates a mature cardiomyocyte at risk of doxorubicin-injury in comparison to a dividing cell-line model (which may be tumour-derived) or an undifferentiated neonatal rat ventricular myocyte model. Of note, we investigated cardiomyocyte death in terminally differentiated adult isolated rat cardiomyocytes in response to a range of concentrations of doxorubicin, and an increase in cell death was noted at, and above, peak plasma concentrations, which became statistically significant from 7.5 μM. We therefore used this concentration in subsequent experiments.

When administered to an isolated, perfused rat heart, a related anthracycline, epirubicin, decreased left ventricular developed pressure (LVDP) within 20 min. IPC was able to decrease the damaging effect of epirubicin [16], but part of this improvement in cardiac function might conceivably be due to protection of the vasculature [27]. The present study demonstrates that IPC can directly protect cardiomyocytes against anthracycline cardiotoxicity.

Doxorubicin is known to modulate the PI3K/Akt and the MAPK/ERK1/2 signalling pathways itself and may contribute to its cardiotoxicity. In in vivo rodent models, after an initial peak in MAPK/ERK1/2 phosphorylation during the first few hours after doxorubicin administration, MAPK/ERK1/2 phosphorylation declines for weeks after the cessation of dosing. This decline is paralleled by lower levels of ERK1/2 mRNA and is accompanied by symptomatic heart failure [7]. Interestingly, in an acute isolated heart model, doxorubicin was found to increase the phosphorylation of Akt and Erk1/2 when applied during the reperfusion phase after ischaemia [28]. In neonatal rat cardiomyocytes, Zhu et al. observed an induction of apoptosis with doxorubicin when ERK1/2 was selectively inhibited [9]. Conversely, activation of the MAPK/ERK1/2 signalling pathway has been suggested to be a key-mediator of the protective effect seen with, for example, the fatty acid oleylethanolamide against doxorubicin toxicity [8]. Since both the PI3K/Akt and MAPK/ERK1/2 cascades are known to be activated by IPC and exert a cardioprotective effect in the acute setting, we explored if sIPC could protect against the cardiotoxicity induced by this drug. Consistent with our hypothesis, sIPC protected cardiomyocytes against a cardiotoxic dose of doxorubicin. Specific inhibition of PI3K/Akt or MAPK/ERK1/2 revealed this protective effect to be dependent on the PI3K/Akt and independent of MAPK/ERK1/2. Our data therefore clarifies the role of signalling cascades of the RISK pathway that may hold protective potential against doxorubicin cardiotoxicity. Interestingly, a previous study has suggested that preconditioning using morphine is able to confer cardioprotection in doxorubicin-induced failing rat hearts via an ERK/GSK-3beta pathway independent of PI3K/Akt [29], which suggests that there may be multiple routes to cardioprotection against anthracyclines.

The mechanism by which PI3K/Akt protected cells against doxorubicin is unclear. PI3K/Akt is known to protect against ischemia-reperfusion injury by reducing ROS and decreasing mPTP opening [23, 30]. Given that ROS have been implicated in anthracycline cardiotoxicity [3, 31], we considered the possibility that PI3K/Akt protected cardiomyocytes by targeting ROS. However, in our in vitro model, doxorubicin toxicity was independent of ROS since a ROS scavenger failed to rescue cardiomyocytes from doxorubicin-induced cell death. This was despite the ROS scavenger protecting cells against H_2_O_2_-induced death. Although ROS are established to be part of the mechanism of doxorubicin-induced toxicity [31], ROS-independent cell-death pathways have previously been implicated in doxorubicin toxicity [32], and moreover, ROS scavengers have not exhibited any clinical benefit against doxorubicin cardiotoxicity [4]. This raises the possibility that IPC can protect against acute anthracycline cardiotoxicity by a mechanism independent of ROS.

Doxorubicin activates additional cell-death mechanisms, including p53 induction and apoptosis secondary to DNA damage [17]. Moreover, doxorubicin may also modulate proteins involved in post-translational modifications such as histone deacetylases [33] and thereby influence apoptotic pathways [18] as well as key homeostatic cellular pathways such as autophagy [34]. Other pathways that have been suggested to mediate doxorubicin-induced cardiomyocyte death include inhibition of the mitochondrial respiratory chain and mitochondrial biogenesis pathways [35]. Doxorubicin-induced cardiotoxicity is therefore likely to be a complex multifactorial process, and IPC may potentially impinge upon any, or all, of these different pathways.

We observed no significant change in the mitochondrial transmembrane potential after up to 24 h treatment with doxorubicin. We further observed no difference in the sensitivity of the mPTP between control group and doxorubicin-treated cardiomyocytes. Surprisingly, cyclosporine A, a known inhibitor of the mPTP, was ineffective in preventing mPTP opening in doxorubicin-treated cardiomyocytes. These results differ from observations reported by other groups, both in in vitro and ex vivo*.* For example, Fisher et al. reported a fall in ΔΨ_m_ in cultured adult mouse ventricular myocytes using the JC-1 dye following 18 h incubation with 1 μM doxorubicin [36]. Similarly, Zhang et al. isolated murine cardiomyocytes 72 h after a large bolus dose of 25 mg/kg doxorubicin intraperitoneally, and using JC-1, they observed a decrease in Δψ_m._ [37]. A possible explanation for these different observations is that we used TMRM rather than JC-1to measure ΔΨ_m_. Aggregate (i.e. red) JC-1 fluorescence is highly sensitive to probe loading concentrations and loading times and may change independently of Δψ_m_ under some circumstances [38]. Using a similar, laser-induced ROS model to ours, Gharanei et al. found that CsA was able to protect cardiomyocytes against mPTP opening in the presence of 1 μM doxorubicin. However, primary adult rat cardiomyocytes were treated with doxorubicin for only 10 min [28], in contrast to our 18-h treatment. Similarly, Montaigne et al. measured the epicardial fluorescence of mitochondrial dye JC-1 in isolated perfused rat hearts following 60 min of 1 μM doxorubicin infusion and found the mitochondrial transmembrane potential dissipated in a time-dependent manner [39].

Importantly, sIPC was ineffective at protecting a cancer cell line against doxorubicin injury in vitro, possibly because it did not activate the cyto-protective PI3K/Akt pathway. This suggests that it may be possible to use a preconditioning stimulus to protect the heart from damage, without concomitantly protecting the tumour being targeted for destruction.

Doxorubicin has been described variously as capable of inflicting necrotic as well as apoptotic cardiomyocyte-death [18]. A potential limitation of our study is that we measured total cell-death as a marker of doxorubicin-induced cardiomyocyte toxicity, without delineating the individual contributions of each death pathway. A further limitation is the relatively high dose of doxorubicin used, which was necessary to reveal cardiomyocyte injury within the 18-h time period. Further studies will be necessary to confirm that similar results are obtained in vivo. The response of cancer cells to doxorubicin may be altered in vivo, and therefore future experiments will be necessary to confirm that IPC does not cause increased survival of cancer cells in vivo. Finally, we recognise the possibility that further replicates may reveal differences we were unable to detect in our Western Blot and ROS scavenger experiments.

## Conclusions and Future Directions

We show that the phenomenon of preconditioning protects cardiomyocytes from doxorubicin cardiotoxicity in vitro. This protection is mediated via the PI3K/Akt component of the RISK pathway. The toxicity of doxorubicin appears independent of ROS production and independent of induction of the mPTP. However, we have not yet determined the precise molecular target of the RISK pathway in protecting the myocytes against doxorubicin-mediated cardiomyocyte death. The protective potential of IPC may be further explored in ex vivo and in vivo animal models.

Given that ischemic preconditioning is an invasive procedure, it is not feasible to administer it to patients undergoing anthracycline treatment. However, it is well established that RISK pathway activation and cardioprotection is also elicited by the application of a preconditioning stimulus to an organ or limb remote from the heart [10]. This intervention, called remote ischemic preconditioning (RIPC), reduces ischemia-reperfusion (IR) injury and decreases peak troponin levels in patients undergoing thrombolysis for ST-elevation myocardial infarction [40]. Future studies will be important to investigate whether RIPC may be effective against anthracycline cardiotoxicity. Indeed, the Effect of Remote Ischaemic Conditioning in Oncology (ERIC-ONC) trial (clinicaltrials.gov NCT 02471885) aims to determine whether RIPC is cardioprotective in adult oncology patients undergoing anthracycline-based chemotherapy treatment [41]. It will be important to confirm that RIPC does not provide a survival advantage to the tumour in situ.
